# The Effect of Chondroitin Sulphate and Hyaluronic Acid on Chondrocytes Cultured within a Fibrin-Alginate Hydrogel

**DOI:** 10.3390/jfb5030197

**Published:** 2014-09-18

**Authors:** Christopher J. Little, William M. Kulyk, Xiongbiao Chen

**Affiliations:** 1 Department of Biomedical Engineering, University of Saskatchewan, Saskatoon, SK S7N 5A9, Canada; E-Mail: cjl519@mail.usask.ca; 2 Department of Anatomy and Cell Biology, University of Saskatchewan, Saskatoon, SK S7N 5E5, Canada; 3 Department of Mechanical Engineering, University of Saskatchewan, Saskatoon, SK S7N 5A9, Canada; E-Mail: xbc719@mail.usask.ca

**Keywords:** osteoarthritis, hyaluronic acid (HA), hydrogel, tissue engineering

## Abstract

Osteoarthritis is a painful degenerative joint disease that could be better managed if tissue engineers can develop methods to create long-term engineered articular cartilage tissue substitutes. Many of the tissue engineered cartilage constructs currently available lack the chemical stimuli and cell-friendly environment that promote the matrix accumulation and cell proliferation needed for use in joint cartilage repair. The goal of this research was to test the efficacy of using a fibrin-alginate hydrogel containing hyaluronic acid (HA) and/or chondroitin sulphate (CS) supplements for chondrocyte culture. Neonatal porcine chondrocytes cultured in fibrin-alginate hydrogels retained their phenotype better than chondrocytes cultured in monolayer, as evidenced by analysis of their relative expression of type II *versus* type I collagen mRNA transcripts. HA or CS supplementation of the hydrogels increased matrix glycosaminoglycan (GAG) production during the first week of culture. However, the effects of these supplements on matrix accumulation were not additive and were no longer observed after two weeks of culture. Supplementation of the hydrogels with CS or a combination of both CS and HA increased the chondrocyte cell population after two weeks of culture. Statistical analysis indicated that the HA and CS treatment effects on chondrocyte numbers may be additive. This research suggests that supplementation with CS and/or HA has positive effects on cartilage matrix production and chondrocyte proliferation in three-dimensional (3D) fibrin-alginate hydrogels.

## 1. Introduction

Osteoarthritis is a term describing the erosion of or damage to articular cartilage, which, due to the inability of cartilage to naturally regenerate itself, poses a serious health concern to millions of people worldwide [1]. Cartilage tissue engineering is a discipline aimed at repairing articular cartilage lesions with artificial cartilage constructs. These constructs consist of a biocompatible three-dimensional (3D) culture environment seeded with a suspension of chondrocytes or mesenchymal chondroprogenitor cells. This culture environment provides initial mechanical support for the cells, as well as providing a micro-environment that supports cell proliferation and the maintenance or acquisition of a differentiated chondrocyte phenotype. However, these artificial cartilage constructs frequently have lower levels of extracellular matrix (ECM) relative to native cartilage [2]. This represents a critical consideration in engineered cartilage, as the mechanical properties of this neocartilage and its integration with the surrounding native cartilage is correlated with the level of ECM deposition [3,4].

There are a number of different methods currently under investigation to enhance this matrix accumulation. For example, the type of 3D culture environment has been shown to affect matrix accumulation, with hydrogels showing higher levels of ECM accumulation and cell proliferation relative to solid scaffolds [5,6]. Alginate hydrogel has been shown to enhance phenotype retention in chondrocytes relative to traditional monolayer culture [7], with the addition of fibrin to alginate enhancing chondrocyte adhesion and proliferation [8,9]. However, although this fibrin-alginate mixed hydrogel has been shown to be superior to alginate, there has not been any direct comparison of chondrogenic phenotype retention in this mixed hydrogel to freshly isolated chondrocytes or chondrocytes cultured in monolayer.

Another method to stimulate ECM production in engineered cartilage constructs is supplementing the hydrogel and culture medium with macromolecules that enhance their phenotype retention. An example of these molecules is the solubilized form of natural cartilage ECM components, such as hyaluronic acid (HA) and chondroitin sulphate (CS). Both of the studies by Akmal *et al.* [10] and Kawasaki *et al.* [11] demonstrated enhanced sulphated glycosaminoglycan (GAG) accumulation and chondrocyte proliferation in hydrogel cultures supplemented with HA relative to non-supplemented hydrogel controls. Similarly, supplementation with CS has been shown to enhance GAG synthesis and cell proliferation in 3D cultures of chondrocytes [12,13].

The objective of the present study was to characterize the effects of HA and CS supplementation on ECM production, chondrocyte proliferation and cartilage-specific gene expression in a 3D fibrin-alginate hydrogel culture system and to determine if these supplement effects are additive.

## 2. Materials and Methods

### 2.1. Isolation of Chondrocytes

Articular cartilage was harvested from the femoral and humeral heads, lateral and medial condyles and patellae of newborn pigs. These piglets were obtained from the Prairie Swine Centre (Saskatoon, SK, Canada) and had died spontaneously no more than 24 h prior to dissection. The cartilage was minced into 1–2 mm fragments using scalpels and subjected to a sequential digestion in which the tissue was incubated for 1 h (37 °C, 5% CO_2_, 100% relative humidity) with 10 mg/mL pronase (Boehringer/Roche, Mississauga, Canada) in Dulbecco’s Modified Eagle Medium (DMEM) (Sigma-Aldrich, Oakville, Canada) supplemented with 0.292 mg/mL glutamine; 100 U/mL penicillin; 100 µg/mL streptomycin, 0.25 µg/mL amphotericin B, 100 µg/mL kanamycin (GAK) and then treated with 2 mg/mL collagenase (Type IA; Sigma-Aldrich) in DMEM supplemented with 10% fetal bovine serum (FBS) and GAK for 4 h at 37 °C with intermittent agitation. The resulting digest was then passed through a 100-µm pore Nitex filter, and the isolated chondrocytes were isolated by centrifugation (10 min, 300 g) and resuspended in DMEM/10% FBS/GAK medium. The cell density was determined using a hemocytometer and adjusted to 5 × 10^7^ cells/mL.

### 2.2. Hydrogel Preparation and Chondrocyte Seeding

Alginate (Sigma-Aldrich) and fibrinogen (Sigma-Aldrich) were dissolved in 150 mM NaCl and 20 mM 4-(2-Hydroxyethyl)piperazine-1-ethanesulfonic acid (HEPES) to stock concentrations of 30 mg/mL alginate and 62.5 mg/mL fibrinogen, then filter sterilized. The 30 mg/mL alginate stock solution was then supplemented with either 0.25 mg/mL HA (from rooster comb; Sigma-Aldrich), 0.25 mg/mL CS (CS-A from bovine trachea; Sigma-Aldrich), both 0.25 mg/mL HA + 0.25 mg/mL CS, or neither additive. Equal aliquots of the fibrinogen and supplemented alginate stock solutions were combined and dispensed in 157 µL aliquots into sterile 1.5-mL microtubes. Then, 19.6 µL of chondrocyte cell suspension (5 × 10^7^ cells/mL) was re-suspended in the fibrinogen/alginate mixture within each tube. Next, 19.6 µL of 100 U/mL thrombin solution (Sigma-Aldrich) were added to each tube followed by incubation at 37 °C for 15 min to catalyze fibrinogen cleavage and fibrin gel formation. The resulting gel was overlaid with 588 µL of 102 mM CaCl_2_ and incubated 20 min at 37 °C to crosslink the alginate. This yielded a hydrogel of 196-µL final volume and final component concentrations of 25 mg/mL fibrinogen, 12 mg/mL alginate, 5 × 10^6^ cells/mL, 10 U/mL thrombin, with final supplement concentrations of 0.1 mg/mL HA and/or 0.1 mg/mL CS. The polymerized hydrogel plugs were transferred into separate wells of 12-well NUNC^®^ tissue culture plates (2.1-cm well diameter; Thermo Fisher Scientific, Ottawa, Canada) and submerged in 2 mL DMEM/10% FBS/GAK medium supplemented with 0.1 mg/mL HA, 0.1 mg/mL CS, both 0.1 mg/mL HA + 0.1 mg/mL CS (HACS) or neither additive. The concentration of 0.1 mg/mL HA employed in our experiments has been reported to enhance matrix production and cell proliferation in 3D chondrocyte cultures [10,11], while 0.1 mg/mL CS supplementation was shown to enhance matrix production [12].

### 2.3. Harvesting of Hydrogels

This was a factorial experiment with one set of hydrogel samples (*n* = 8 for each supplement treatment group) harvested after 1 week of culture, another harvested after 2 weeks (*n* = 6 for each treatment) and 5 non-supplemented hydrogels being harvested immediately (0 days). The mean wet weight of the hydrogels after 1 week of culture was 213 mg (±standard deviation of 15.6) and was not significantly different among the various treatment groups. The harvested hydrogels were rinsed in Hank’s saline for 10 min, after which they were incubated at 37 °C with 471 µL of hydrogel dissolution solution (90 mM NaCl, 55 mM sodium citrate, 30 mM EDTA, 0.1 mM Tris HCl, pH 7.5, 0.2 mM CaCl_2_, 0.2 mg/mL proteinase K (Invitrogen Life Technologies, Burlington, Canada) until the gels were completely dissolved. A 30.4-µL aliquot of this digest was removed and mixed with 70.6 µL triple-distilled water, then stored at 4 °C for later use in sulphated GAG quantification, as described below. The remaining hydrogel digest was centrifuged (5 min, 4 °C, 300 g), the supernatant removed, and the pelleted cells were resuspended in 110 µL of Hank’s saline. A 10-µL aliquot of this cell suspension was mixed with 160 µL of Hank’s saline and frozen for the Hoechst DNA quantification assay (*n* = 5, non-supplemented initial hydrogels; *n* = 7, for Week 1; and *n* = 5, for Week 2; for each of the four treatment groups). For the Week 1 samples only, the remaining 100 µL of cell suspension were mixed with 300 µL of TRIzol^®^ LS reagent (Invitrogen Life Technologies). The cells were lysed by repeated aspiration through a 27-gauge syringe needle and frozen at −80 °C for later RNA analysis.

One hydrogel sample from each treatment group at the 1-week and 2-week time points was used for histological sectioning and staining. Hydrogels were rinsed in Hank’s saline, then immersed in formaldehyde solution (3.7% formaldehyde in phosphate buffered saline (PBS) supplemented with Mg^2+^ and Ca^2+^) for 30 min to fix the cells. The fixed hydrogel samples were soaked overnight in a sucrose cryopreservation solution (30% sucrose in PBS supplemented with Mg^2+^ and Ca^2+^), embedded in Optimal Cutting Temperature Compound (Tissue-Tek) and frozen at −80 °C prior to cryostat sectioning and immunocytochemical staining (see Section 2.7).

### 2.4. Sulphated GAG Analysis

Some hydrogel samples (*n* = 7 for 1-week cultures; *n* = 5 for 2-week cultures of each treatment group) were used for biochemical analysis of total sulphated GAG content. The 1,9-dimethylmethylene blue (DMMB) assay used for sulphated GAG analysis was adapted from a combination of several protocols found in the literature [10,14,15,16] and modified for use with alginate by adjusting the pH of the dye to 1.5. Briefly, 21 mg of DMMB (Sigma-Aldrich) were dissolved in 5 mL absolute ethanol. Then, 2 g of sodium formate was added, and this solution was mixed with 800 mL of triple-distilled water. Formic acid (95%, Sigma-Aldrich) was added to the solution until the pH was 1.5 and distilled water was added to a final volume of 1 L. A set of GAG standards was created by mixing seeded fibrin-alginate hydrogels (as in Section 2.2) supplemented with known amounts of CS (0–100 µg/mL final concentration).

A 48-µL aliquot of diluted hydrogel digest (see Section 2.3) was added to 300 µL of the DMMB dye and incubated at room temperature for 1 h. Thereafter, the samples and standards were centrifuged (15 min, 16 °C, 11,500 g), and 280 µL of each supernatant were pipetted into a 96-well plate. The absorbance was read at 600 nm using a spectrophotometric plate reader (SLT Spectra; Tecan, Morrisville, ND, USA).

A control experiment was performed to ensure that the exogenous CS incorporated into CS supplemented hydrogels did not create an interfering background signal in our DMMB assay for sulphated GAG accumulation. We measured no significant difference in initial DMMB absorbance readings obtained from CS supplemented hydrogels relative to non-supplemented hydrogels (*data not shown*). This confirmed that the amount of solubilized exogenous CS remaining in hydrogels after a 10-min Hank’s saline rinse (see Section 2.3) was not sufficient to interfere with our DMMB assay for sulphated GAG accumulation.

### 2.5. DNA Quantification Assay

Some samples were subjected to fluorometric DNA quantification following a modification of the method of Labarca * et al.* [17]. Cells were released from solubilized hydrogels (see Section 2.3) and sonicated. A 70-µL aliquot of the resulting sonicate was mixed with 30 µL of 4 µg/mL Hoechst 33258 dye (Sigma-Aldrich) and 100 µL of 2× buffer (0.1 M NaH_2_PO_4_, 4 M NaCl, pH 7.4). Samples were then loaded into a 96-well black microtiter plate and fluorescence was quantified using a Fluorolite 1000 (Dynex Technologies, Worthing, UK) plate reader at an excitation wavelength of 365 nm and an emission wavelength of 458 nm. The samples were run alongside a set of DNA standards (0–4 µg/100 µL chicken DNA) for absolute quantification of DNA content.

### 2.6. RNA Analysis by Real-Time Quantitative PCR (qPCR)

Cells released from solubilized hydrogels were lysed in TRIzol^®^ LS reagent, as detailed in Section 2.3. The lysate was centrifuged (10 min, 12,000 g, 4 °C) to pellet any insoluble debris. The supernatant was mixed with 80 µL of chloroform, incubated at room temperature for 5 min, then centrifuged (15 min, 12,000 g, 4 °C) to separate the aqueous and organic phases. The upper aqueous phase was drawn off and mixed with an equal volume of 70% ethanol. This solution was then added to a HiBind RNA column from an E.Z.N.A™ Total RNA Kit (Omega, BioTek, Norcross, GA, USA), and the manufacturer’s protocol for RNA isolation was followed.

After RNA isolation, the A_260_/A_280_ ratio was measured to check for phenol or protein contamination. Then, cDNA was synthesized from this RNA using the QuantiTect^®^ Reverse Transcription Kit (Qiagen, Toronto, Canada) following the manufacturer’s protocol. The cDNA was used as a template for real-time quantitative polymerase chain reaction (qPCR) using a Fermentas Maxima^®^ SYBR Green reagents system (Thermo Fisher Scientific, Ottawa, Canada) in accordance with the manufacturer’s protocol. Amplifications were performed on an MJ Mini thermal cycler equipped with a Bio-Rad MiniOpticon fluorescence detection module. The PCR cycle parameters were 95 °C for 15 s, 65.8 °C for 30 s and 72 °C for 30 s. Expression levels of four types of gene transcripts were analyzed: the mRNAs that code for collagen type I (col1α1), collagen type II (col2α1), aggrecan and glyceraldehyde 3-phosphate dehydrogenase (GAPDH) as a reference gene. The sequences of all qPCR primers used are described in Table 1.

**Table 1:** List of primers used for qPCR.

Gene	GenBank Accession Number	Forward Sequence (5'-3')	Reverse Sequence (5'-3')	Amplicon Size	Optimal Annealing Temperature Range (°C)
GAPDH	DQ403065	5'-ACGGCAAGTTC CACGGCACAGT-3'	5'-GTTGGCGGGAT CTCGCTCCTGG-3'	95	53.0–69.0
Collagen Type II	AF201724	5'-CAAAGATGGCGA GACAGGTGCT-3'	5'-GAAGTCCCTGGA AGCCAGATGGC-3'	104	53.0–65.8
Collagen Type I	AF201723	5'-GACGCACGGCCA AGAGGAGG-3'	5'-CTGGCAGGGCAC GGGTTTCC-3'	130	59.5–65.8
Aggrecan	NM_001164652	5'-GCTTCCGAGGTGT CTCGGCG-3'	5'-TCGTCTCCTCGC CCACAGGG-3'	141	56.4–69.0

For each gene, a temperature gradient and serial dilution were run to determine the optimum PCR annealing temperature and PCR amplification efficiency, respectively. To ensure that each primer pair only amplified its specific target gene sequence, both gel electrophoresis and melt-curve analysis were performed on the PCR products. No template controls (NTCs; *i.e.*, PCR reaction mixtures lacking a cDNA template) were run alongside the samples to check for the amplification of primer-dimers or contamination of the reagents. In addition, 6 samples were randomly chosen for “no reverse transcriptase controls” (NRTs) to check for genomic DNA contamination in the isolated RNA. For both the NTCs and NRTs, if amplification took place more than 5 cycles after the lowest expressed transcript in the experimental samples, it was considered negligible, as recommended by Bustin *et al.* [18]. Finally, 6 randomly chosen samples were analyzed on a Bio-Rad Experion microfluidic electrophoresis system to verify the integrity of the input RNA used for cDNA synthesis and qPCR amplification.

### 2.7. Immunostaining

Representative hydrogels were cryogenically sectioned using a HM550 cryostat (Thermo Fisher Scientific, Ottawa, Canada) at a thickness of 30 µm. The sections were subjected to immunostaining to detect the presence of collagen types I and II. First, the sections were washed 2× with PBS containing 0.01% Tween 20 (PBST), for 5 min per wash. The sections were then incubated in a blocking solution of PBST containing 5% sheep serum (Sigma-Aldrich) for 1 h. The blocking solution was replaced with the diluted primary antibody and allowed to incubate at room temperature for 1 h. The primary antibody solution for collagen type II (hybridoma supernatant II-II6B3; Developmental Studies Hybridoma Bank, Iowa City, IA, USA) was diluted 1:25 with PBST containing 5% sheep serum, while the collagen type I monoclonal antibody (Catalog #2456, Sigma-Aldrich) was diluted 1:200. Following the primary antibody treatment, the sections were washed 4× with PBST (10 min per wash). Then, the sections were incubated for 1 h at room temperature in a 1:200 dilution of alkaline phosphatase-conjugated goat anti-mouse secondary antibody (400 µg/mL stock; Santa Cruz Biotechnology, Dallas, TX, USA) in PBST containing 5% sheep serum. Then, the sections were washed 3× with PBST containing 0.5 mg/mL levamisole (10 min per wash), followed by two 15 min washes in 100 mM Tris HCl, pH 9.5, 50 mM MgCl_2_, 100 mM NaCl, 1 mg/mL Triton X-100, 0.5 mg/mL levamisole (NTMT). Finally, the sections were incubated overnight at room temperature in colour development solution (NTMT supplemented with 0.34 mg/mL nitroblue tetrazolium chloride and 0.175 mg/mL 5-bromo-4-chloro-3-indolylphosphate). The colour development reaction was stopped by three, 5-min washes in PBST. As a negative control, a parallel section was treated as above, except that the primary antibody incubation step was omitted.

### 2.8. Statistics

For both the DMMB and the Hoechst assays, a mixed-effects linear model, with the unsupplemented hydrogels as the intercept, was used to identify statistical interactions between the HA and CS treatments that would be indicative of additive, synergistic or antagonistic biological effects. In addition, this allowed for gels that were fabricated at equivalent time points following component mixing to be blocked together in the statistical analysis in order to better resolve significant differences between the non-supplemented hydrogels and the HA, CS and HACS treatment groups.

Analysis of variance (ANOVA) together with the Tukey’s honest significant differences post hoctest was used to determine the change in total cellular DNA in untreated hydrogel cultures over the 2-week period of culture All of the above parametric tests were performed using R^©^ (version 2.10.1) software [19].

The Relative Expression Software Tool (REST) [20] was used for the non-parametric analysis of qPCR data on collagen I, collagen II aggrecan and GAPDH gene transcript levels in hydrogel cultures, monolayer cultures and freshly isolated chondrocytes.

## 3. Results

**Figure 1:**
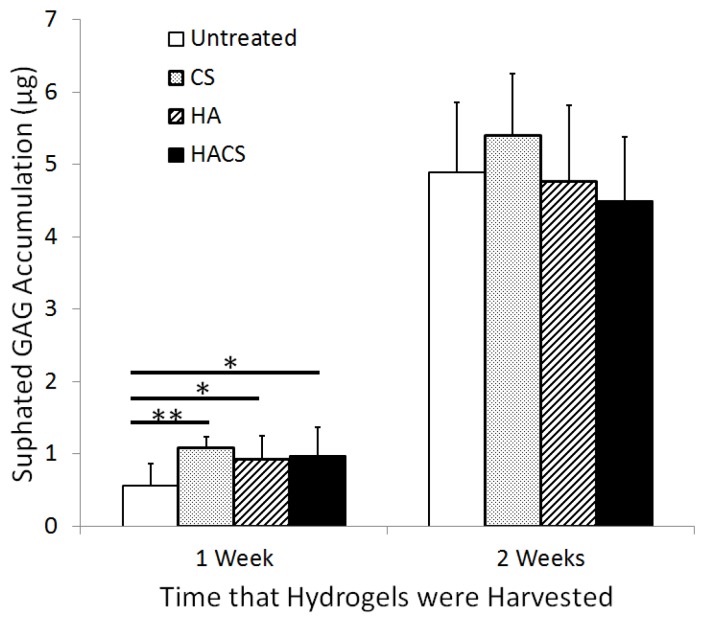
The effects of hyaluronic acid (HA), chondroitin sulphate (CS) or combined hyaluronic acid and chondroitin sulphate (HACS) supplementation on the levels of sulphated glycosaminoglycan (GAG) synthesis in fibrin-alginate hydrogels cultured for one week (*n* = 7 for each treatment) and two weeks (*n* = 5 for each treatment). Untreated hydrogel cultures were not supplemented with either HA or CS. Bar heights represent mean values with error bars of one standard deviation. Asterisks indicate values that are significantly different from the untreated hydrogel cultures (** *p* < 0.01; * *p* < 0.05).

The DMMB GAG quantification assay was used to quantify matrix production (indicated by sulphated GAG levels) in CS, HA and HACS supplemented hydrogel cultures relative to non-supplemented hydrogel cultures. This assay (Figure 1) showed significantly higher levels of sulphated GAG accumulation in the HA, CS and HACS supplemented hydrogels relative to non-supplemented hydrogels after one week of culture, but no significant difference in sulphated GAG abundance after two weeks of culture. The effects of the HA and CS treatments on GAG production were not additive, as hydrogels treated with both HA and CS in combination (HACS) were no higher in sulphated GAG accumulation than hydrogels supplemented with either HA or CS alone (Figure 1). This was confirmed by statistical analysis using a mixed-effects linear model.

**Figure 2:**
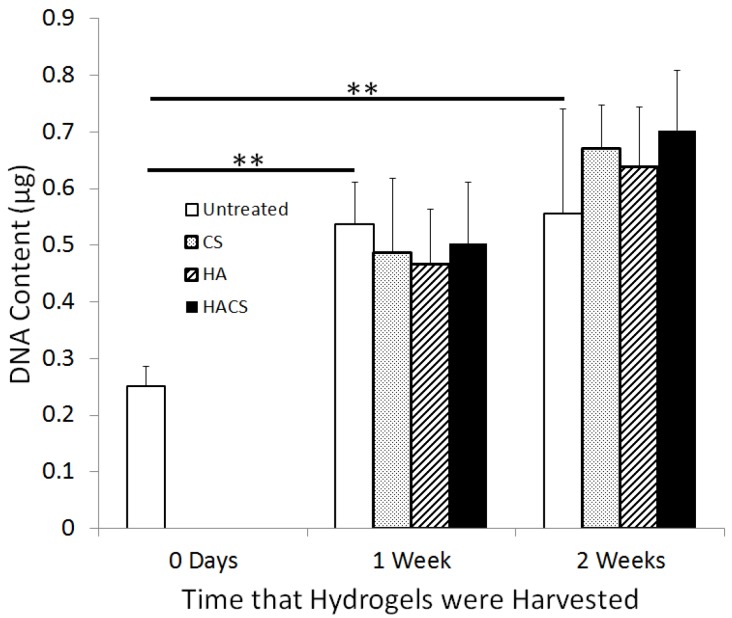
The effects of HA, CS and HACS supplementation on total cellular DNA levels (an indicator of relative chondrocyte numbers) in fibrin-alginate hydrogels that were cultured for one week (*n* = 7 for each treatment), or two weeks (*n* = 5 for each treatment), or harvested immediately (0 days) after cell seeding (*n* = 5). Untreated hydrogels were not supplemented with either HA or CS. Error bars represent one standard deviation from the mean. Asterisks indicate a significant increase in total cellular DNA levels in untreated hydrogels after one and two weeks of culture compared to the beginning of culture (0 days). (** *p* < 0.01).

The Hoechst DNA assay was used to quantify relative cell numbers within the hydrogel cultures. Within the non-supplemented hydrogels, there was roughly a two-fold increase in total cellular DNA during the first week of culture, but the cell population did not increase any further during the second week of culture (Figure 2). After one week of culture, the HA, CS and HACS supplemented hydrogels had slightly lower mean cellular DNA than non-supplemented control hydrogels, although the decrease was not significant. In contrast, by two weeks of culture, the HA, CS and HACS supplemented hydrogels demonstrated modestly higher mean cellular DNA levels relative to untreated controls. Statistical analysis using a mixed effects linear model, which allowed for statistical pairing of hydrogel cultures fabricated at the same time, indicated that the increase in cellular DNA levels was significant in CS and HACS supplemented hydrogels relative to non-supplemented hydrogel controls. Furthermore, statistical analysis using the mixed effects linear model suggests that the HA and CS treatments had additive effects on cellular DNA accumulation in the two-week hydrogel cultures.

**Figure 3:**
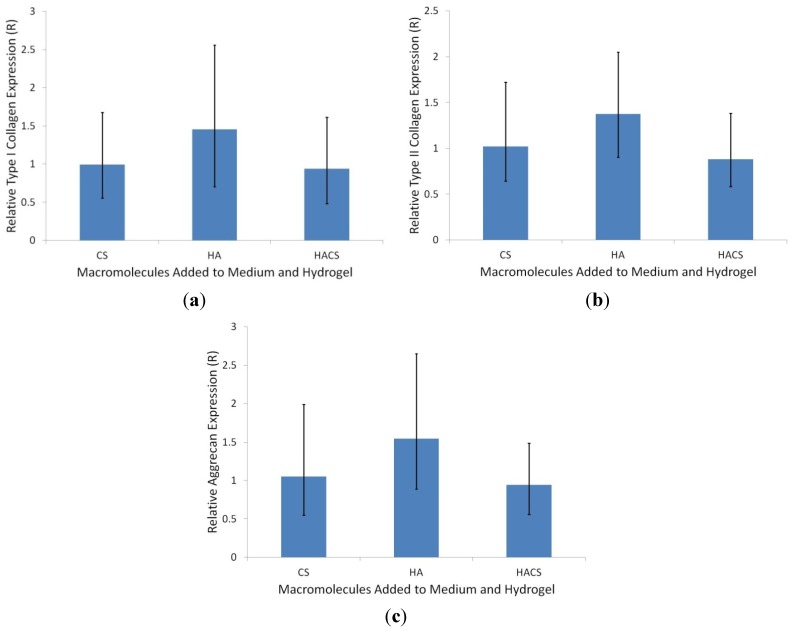
Graphs depicting the relative expression levels (*R*) of the mRNAs that code for (**a**) collagen type I, (**b**) collagen type II and (**c**) aggrecan in hydrogels supplemented with CS (*n* = 7), HA (*n* = 6) and HACS (*n* = 7), expressed as the ratio of their corresponding mRNA levels in non-supplemented hydrogels, which are assigned an *R* value of one. The bar heights represent the median ± standard error as calculated by the Relative Expression Software Tool (REST) statistical analysis. (Note: the collagen I, collagen II and aggrecan gene expression values shown are normalized against the mRNA level for the constitutively expressed GAPDH transcript. There was no significant difference in GAPDH RNA levels between the HA, CS, HACS and non-supplemented hydrogel treatment groups; *data not shown*).

Quantitative real-time PCR (qPCR) was used to determine the expression levels of mRNAs that code for collagen type I (col1α1), collagen type II (col2α1), aggrecan and GAPDH. The collagen I, collagen II and aggrecan gene transcript levels in HA, CS and HACS supplemented hydrogels were normalized against the mRNA level for the constitutively expressed GAPDH reference gene and expressed as a ratio of the corresponding target gene expression in non-supplemented control hydrogels (Figure 3). Statistical analysis of the qPCR data using REST software revealed no significant up- or down-regulation of collagen I, collagen II or aggrecan gene expression when comparing the three different supplement treatments to the untreated control group.

**Figure 4:**
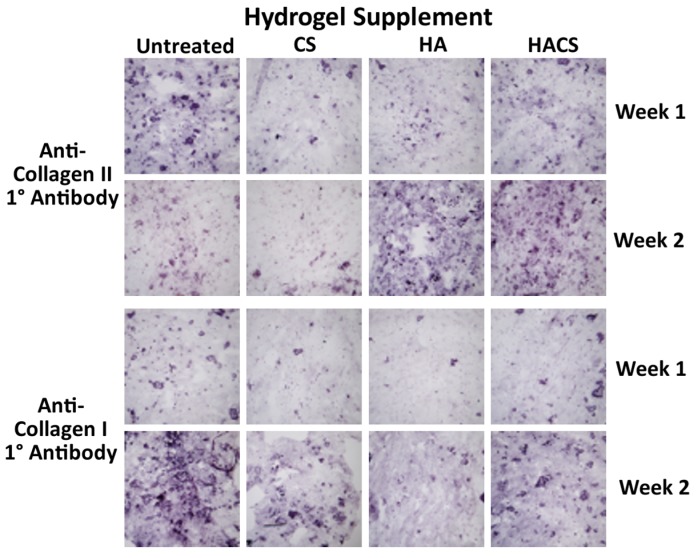
Immunostaining of cryogenic sections of hydrogels supplemented with CS, HA or HACS. Non-supplemented hydrogels (untreated) served as controls. Staining revealed that there was an accumulation of collagen types I and II proteins over time in all treatment groups. All photomicrographs were taken at equivalent magnification on a Leitz DM-RB compound microscope (10× objective lens) equipped with a Sony DSC-V3 camera.

Representative hydrogel cultures from the HA, CS, HACS and non-supplemented treatment groups were sectioned with a cryostat and immunocytochemically stained with antibodies specific for collagen type I and collagen type II proteins. As seen in Figure 4, both collagen I and collagen II proteins were detected in the hydrogels from all four treatment groups at one and two weeks of culture. This confirmed that the collagen I and II mRNAs detected by qPCR were indeed being translated into protein. However, we were unable to perform quantitative comparisons on the relative abundance of collagen I or collagen II proteins between the different supplement treatment groups due to variability in the quality of histological sections between individual hydrogel samples, as well as considerable variability in staining intensity across different areas from the same sample.

## 4. Discussion

A major consideration in the engineering of artificial cartilage tissue constructs is developing an optimized culture environment that supports the survival and proliferation of embedded chondrocytic cells, while also preserving the chondrocyte phenotype and maintaining the biosynthesis of cartilage-specific ECM components. Numerous studies have shown that propagation of chondrocytes in monolayer cultures on standard tissue culture dishes leads to their gradual dedifferentiation, which is characterized by a switch from collagen II to collagen I synthesis, decreased aggrecan secretion and the adoption of a fibroblastic morphology [21]. In contrast, embedding chondrocytes in 3D hydrogels composed of various natural polymers or synthetic polymers helps sustain their normal patterns of gene expression and cartilage matrix secretion [5,6,7,22]. Perka *et al.* [9] demonstrated the advantages of a fibrin-alginate hydrogel scaffold system, which provided a better environment for long-term chondrocyte culture than hydrogels composed of fibrin or alginate alone. Consistent with these earlier reports, we observed that neonatal porcine chondrocytes propagated in monolayer cultures exhibited a progressive decline in collagen II and aggrecan gene transcript expression over a 6–12-day period of culture and a coincident rise in collagen I expression (Supplemental Figure S1). In contrast, chondrocytes embedded in a fibrin-alginate hydrogel demonstrated a significantly higher ratio of collagen II to collagen I mRNA expression in comparison to chondrocytes cultured in monolayer (Supplemental Figure S2).

The purpose of the present study was to examine the effects of supplementing the fibrin-alginate hydrogel with exogenous HA and CS on the parameters of chondrocyte population growth, ECM production and cartilage-specific gene expression. Moreover, we wished to determine whether supplementation of the hydrogel with both CS and HA in combination (HACS) would have additive effects on these aspects of chondrocyte behavior.

We found that supplementation with either CS, HA or HACS elevated levels of matrix sulphated GAG production in chondrocytes cultured within a fibrin/alginate hydrogel. This is consistent with earlier reports that exogenous HA and CS can enhance ECM deposition in chondrocyte cultures [10,11,13]. Interestingly, the stimulatory effects of CS and HA on sulphated GAG accumulation that we observed were only evident during the first week of culture. It is possible that the effects of exogenous CS and HA become negligible by two weeks of culture as a result of the increasing deposition of newly synthesized aggrecan, an endogenous cartilage-specific chondroitin sulphate proteoglycan, by chondrocytes embedded in the fibrin-alginate hydrogel. A previous study by van Susante *et al.* [13] similarly showed that matrix production slowed with progressive GAG accumulation in a chondroitin sulphate-collagen I scaffold system for chondrocyte culture.

Contrary to our expectations, we found that the stimulatory effects of CS and HA supplementation on sulphated GAG production in fibrin-alginate hydrogels were not additive. It is possible that the 1-mg/mL concentration of CS and HA supplements employed in our experiments were each sufficient by themselves to stimulate matrix GAG synthesis maximally.

Supplementation with CS, alone or in combination with HA, also had positive effects on chondrocyte population growth within the fibrin-alginate hydrogels. By two weeks of culture, both CS and HACS supplemented hydrogels displayed significantly higher total cellular DNA accumulation than non-supplemented hydrogels. Treatment with HA alone caused no statistically significant increase in the mean cellular DNA level. However, a combination of HA supplementation with CS had additive effects on chondrocyte proliferation that were greater than the effects of CS supplementation alone. It is worth noting that the stimulatory effects of CS and HACS supplements on chondrocyte population growth were not observed during the initial week of hydrogel culture. Similar trends were reported in studies by van Susante *et al.* [13] and Akmal *et al.* [10], who observed temporally delayed effects of HA and CS treatments on the proliferation of cultured chondrocytes.

Our study also examined whether supplementation of fibrin-alginate hydrogels with CS and/or HA affected expression levels of the cartilage-specific aggrecan and collagen II gene transcripts or the mRNA level for collagen I, a marker for dedifferentiated chondrocytes. However, our qPCR data revealed no statistically significant effects of either CS, HA or HACS supplementation on the levels of expression of any of these target genes. This suggests that the observed stimulatory effects of the CS and HA supplementation on GAG accumulation might be due to some post-transcriptional influence on the expression of sulphated matrix proteoglycans, like aggrecan. In agreement with our findings, an earlier study by Nishimoto *et al.* [23] reported that treatments with CS and low molecular weight HA had no significant effect on collagen II and aggrecan gene transcript levels in a 3D chondrocyte culture system.

Consistent with the expression of both collagen II mRNA and collagen I mRNA transcripts in our hydrogel cultures, immunocytochemical staining confirmed that both type II and type I collagen proteins were deposited into the fibrin-alginate gel. For both collagen II and collagen I, the immunostaining appeared to be predominantly pericellular. As such, the presence of both collagen II and collagen I proteins in the fibrin-alginate hydrogels suggests that the embedded cells may be a mixture of phenotypically differentiated chondrocytes together with some chondrocytes undergoing dedifferentiation. Unfortunately, we could not determine whether the CS, HA or HACS supplementation treatments induced any quantitative changes in collagen II or collagen I protein abundance, due to variability in the quality of the histological sections obtained from different hydrogel samples, as well as the variable immunostaining intensity in different areas of the same specimen.

Our present study has a number of limitations that should be addressed in future investigations. We tested only one concentration of CS and HA supplementation (0.1 mg/mL) in our fibrin-alginate hydrogels, whereas optimal effects on chondrocyte behavior might occur at higher or lower doses. Akmal * et al.* [10] demonstrated that HA dosage influenced both matrix accumulation and chondrocyte proliferation, and it is likely that the effects of CS and HA in combination are also dose-dependent. Moreover, in addition to CS and HA, there are numerous other macromolecules (e.g., collagen II) that may be tested alone or in combination as candidate supplements for improving the quality of engineered cartilage constructs, together with stimulation by soluble growth factors, such as bone morphogenetic proteins (BMPs) and transforming growth factors-beta (TGF-βs) that elevate cartilage-specific gene transcription and matrix production. In future studies, it will be important to perform mechanical testing [4] on the hydrogels after prolonged culture in order to determine the effects of supplementation treatments on the load-bearing ability of the constructs. Developing optimized supplementation regimens for a 3D chondrocyte culture that are effective at preserving a differentiated chondrocyte phenotype and assembling an ECM with properties resembling that of natural cartilage matrix is crucial to engineering implantable cartilage tissue substitutes capable of functional integration with surrounding native cartilage. A further challenge is to fabricate artificial cartilage constructs that mimic the stratified zonal architecture of natural articular cartilage with heterogeneous layer-specific biological and mechanical properties [22,24].

## 5. Conclusions

Our study demonstrates that CS and HA supplements, either alone or in combination, enhance matrix GAG accumulation and cell proliferation by articular chondrocytes embedded in 3D fibrin-alginate hydrogels. The stimulatory effects of CS and HA on chondrocyte population growth were additive and became evident following two weeks of *in vitro* culture. By contrast, the stimulatory effects CS and HA on sulphated GAG production were non-additive and were exhibited only during the first week of culture. A critical consideration in developing improved scaffold systems for cartilage tissue engineering is fabricating an artificial microenvironment with optimized macromolecular composition that maintains the differentiated phenotype of embedded chondrocytes and encourages their robust biosynthesis of a cartilage-characteristic ECM.

## Supplementary Files

Supplementary File 1

## References

[1] Lawrence R.C., Felson D.T., Helmick C.G., Arnold L.M., Choi H., Deyo R.A., Gabriel S., Hirsch R., Hochberg M.C., Hunder G.G. (2008). Estimates of the prevalence of arthritis and other rheumatic conditions in the United States part II. Arthritis Rheum..

[2] Woodfield T.B.F., van Blitterswijk C.A., de Wijn J., Sims T.J., Hollander A.P., Riesle J. (2005). Polymer scaffolds fabricated with pore-size gradients as a model for studying the zonal organization within tissue-engineered cartilage constructs. Tissue Eng..

[3] Obradovic B., Martin I., Padera R.F., Treppo S., Freed L.E., Vunjak-Novakovic G. (2001). Integration of engineered cartilage. J. Orthop. Res..

[4] Little C.J., Bawolin N.K., Chen X.B. (2011). Mechanical properties of natural cartilage and tissue engineered constructs. Tissue Eng. B Rev..

[5] Gong Y., He L., Li J., Zhou Q., Ma Z., Gao C., Shen J. (2006). Hydrogel-filled polylactide porous scaffolds for cartilage tissue engineering. J. Biomed. Mater. Res. B.

[6] Lee C.R., Grad S., Gorna K., Gogolewski S., Goessl A., Alini M. (2005). Fibrin-polyurethane composites for articular cartilage tissue engineering: A preliminary analysis. Tissue Eng..

[7] Lee D.A., Reisler T., Bader D.L. (2003). Expansion of chondrocytes for tissue engineering in alginate beads enhances chondrocytic phenotype compared to conventional monolayer techniques. Acta Orthop. Scand..

[8] Lindenhayn K., Perka C., Spitzer R., Heilmann H., Pommerening K., Mennicke J., Sittinger M. (1999). Retention of hyaluronic acid in alginate beads: Aspects for *in vitro* cartilage engineering. J. Biomed. Mater. Res. A.

[9] Perka C., Spitzer R.S., Lindenhayn K., Sittinger M., Schultz O. (2000). Matrix-mixed culture: New methodology for chondrocyte culture and preparation of cartilage transplants. J. Biomed. Mater. Res. A.

[10] Akmal M., Singh A., Anand A., Kesani A., Aslam N., Goodship A., Bentley G. (2005). The effects of hyaluronic acid on articular chondrocytes. Bone Joint J..

[11] Kawasaki K., Ochi M., Uchio Y., Adachi N., Matsusaki M. (1999). Hyaluronic acid enhances proliferation and chondroitin sulfate synthesis in cultured chondrocytes embedded in collagen gels. J. Cell. Physiol..

[12] Bassleer C.T., Combal J.P., Bougaret S., Malaise M. (1998). Effects of chondroitin sulfate and interleukin-1 beta on human articular chondrocytes cultivated in clusters. Osteoarthr. Cartil..

[13] Van Susante J.L.C., Pieper J., Buma P., van Kuppevelt T.H., van Beuningen H., van der Kraan P.M., Veerkamp J.H., van den Berg W.B., Veth R.P.H. (2001). Linkage of chondroitin-sulfate to type I collagen scaffolds stimulates the bioactivity of seeded chondrocytes *in vitro*. Biomaterials.

[14] Enobakhare B.O., Bader D.L., Lee D.A. (1996). Quantification of sulfated glycosaminoglycans in chondrocyte/alginate cultures, by use of 1,9-dimethylmethylene blue. Anal. Biochem..

[15] Lee C.S.D., Gleghorn J.P., Won Choi N., Cabodi M., Stroock A.D., Bonassar L.J. (2007). Integration of layered chondrocyte-seeded alginate hydrogel scaffolds. Biomaterials.

[16] Williams G.M., Klein T.J., Sah R.L. (2005). Cell density alters matrix accumulation in two distinct fractions and the mechanical integrity of alginate-chondrocyte constructs. Acta Biomater..

[17] Labarca C., Paigen K. (1980). A simple, rapid, and sensitive DNA assay procedure. Anal. Biochem..

[18] Bustin S.A., Benes V., Garson J.A., Hellemans J., Huggett J., Kubista M., Mueller R., Nolan T., Pfaffl M.W., Shipley G.L. (2009). The MIQE Guidelines: Minimum Information for Publication of Quantitative Real-Time PCR Experiments. Clin. Chem..

[19] R Development Core Team (2009). R: A Language and Environment for Statistical Computing.

[20] Pfaffl M.W., Horgan G.W., Dempfle L. (2002). Relative expression software tool (REST©) for group-wise comparison and statistical analysis of relative expression results in real-time PCR. Nucleic Acids Res..

[21] Cheng T., Maddox N.C., Wong A.W., Rahnama R., Kuo A.C. (2012). Comparison of gene expression patterns in articular cartilage and dedifferentiated articular chondrocytes. J. Orthop. Res..

[22] Izadifar Z., Chen X., Kulyk W. (2012). Strategic design and fabrication of engineered scaffolds for articular cartilage repair. J. Funct. Biomater..

[23] Nishimoto S., Takagi M., Wakitani S., Nihira T., Yoshida T. (2005). Effect of chondroitin sulfate and hyaluronic acid on gene expression in a three-dimensional culture of chondrocytes. J. Biosci. Bioeng..

[24] Nguyen L.H., Kudva A.K., Saxena N.S., Roy K. (2011). Engineering articular cartilage with spatially-varying matrix composition and mechanical properties from a single stem cell population using a multi-layered hydrogel. Biomaterials.

