# Supplementary material for: The Effect of Chondroitin Sulphate and Hyaluronic Acid on Chondrocytes Cultured within a Fibrin-Alginate Hydrogel
Source: J Funct Biomater. 2014 Sep 18;5(3):197–210. doi: 10.3390/jfb5030197 (PMC4192613; doi:10.3390/jfb5030197)
Supplement: Supplementary File 1 [file jfb-05-00197-s001.pdf]

OPEN ACCESS
Journal of
Functional Biomaterials
ISSN 2079-4983
www.mdpi.com/journal/jfb

Supplementary Materials

Figure S1. Results from qPCR analysis comparing the relative expression levels of the mRNAs that code for (**a**) collagen I, (**b**) collagen II, and (**c**) aggrecan by chondrocytes cultured for 7 days in non-supplemented 3D fibrin-alginate hydrogels (n = 7), *vs.* chondrocytes cultured in monolayer for either 6 days (n = 3) or 12 days (n = 3). In these graphs, the collagen and aggrecan RNA levels for both the hydrogel and monolayer cultures are expressed as a ratio of the corresponding RNA levels in freshly isolated chondrocytes, which were assigned an R value of 1. Bar heights represent the median value ± standard error as determined by REST statistical analysis.

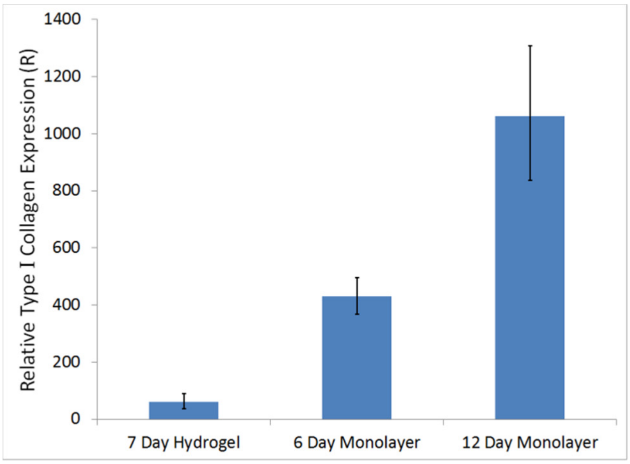

(a)

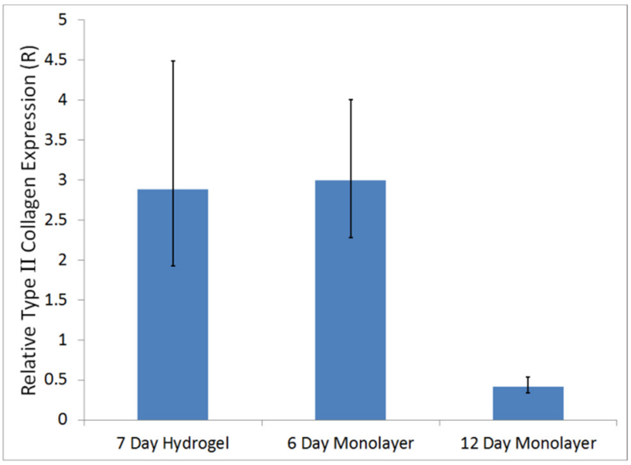

(b)

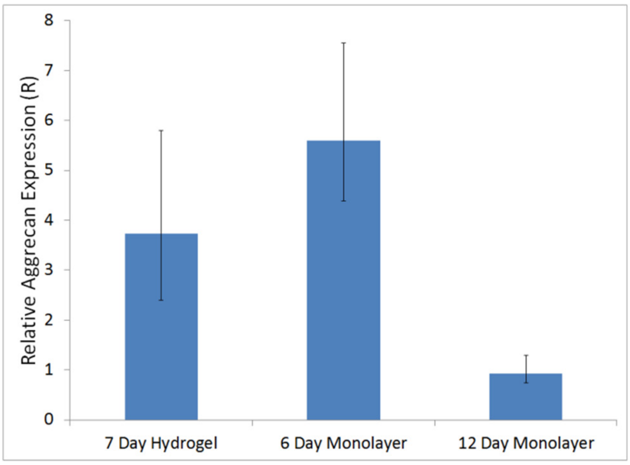

(c)

Figure S2. Results from qPCR analysis comparing the ratio of collagen II RNA to collagen I RNA expression in freshly isolated chondrocytes (*n* = 3), chondrocytes cultured for 7 days in 3D fibrin-alginate hydrogels (*n* = 7), and chondrocytes cultured in monolayer for either 6 days (*n* = 3) or 12 days (*n* = 3). Bar heights represents the median value ± standard error as calculated by REST statistical analysis. A high collagen II/collagen I RNA expression ratio is characteristic of phenotypically differentiated chondrocytes, whereas dedifferentiated chondrocytes exhibit low collagen II/collagen I expression ratios. (In this logarithmic graph, *R* = 1 if collagen II and collagen I gene expression levels are equal. A value below 1 indicates that the collagen II RNA expression is lower than collagen I expression.).

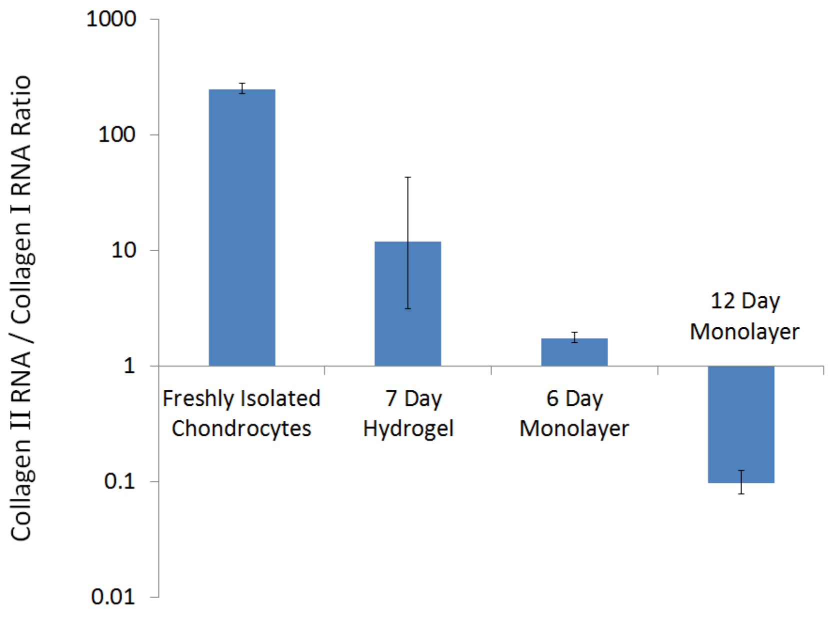

© 2014 by the authors; licensee MDPI, Basel, Switzerland. This article is an open access article distributed under the terms and conditions of the Creative Commons Attribution license (http://creativecommons.org/licenses/by/3.0/).
